# Subjective Job Insecurity During the COVID-19 Pandemic in Italy

**DOI:** 10.1007/s40797-022-00209-z

**Published:** 2022-09-15

**Authors:** Nunzia Nappo, Damiano Fiorillo, Giuseppe Lubrano Lavadera

**Affiliations:** 1 Department of Political Science, University of Naples Federico II, Naples, Italy; 2 Department of Business and Economics, Parthenope University of Naples, Naples, Italy; 3 Dipartimento per la Ricerca, Link Campus University, Rome, Italy

**Keywords:** Subjective job insecurity, COVID-19 pandemic, Government social policy, Remote work, Italy, C21, C25, H53, J01, J81

## Abstract

This article studies subjective job insecurity in Italy during the COVID-19 pandemic, which caused structural, economic and policy changes. It employs data drawn from the “Extraordinary Survey on Italian Families in 2020” released by the Bank of Italy in 2021. The work estimates a zero one inflated beta model (ZOIB). The main results show that (1) worsening household economic conditions is associated with an increase in householder workers subjective job insecurity; (2) receiving a wage guarantee fund in 2019 is associated with an increase in subjective job insecurity; and (3) increasing the number of people working from home within the household is associated with an increase in subjective job insecurity. Moreover, having a temporary contract and working in the private sector is associated with an increase in subjective job insecurity.

## Introduction

In recent decades, processes of globalization, the implementation of skill-biased methods of production and, in some countries, the introduction of new typologies of job contracts have been producing continuous changes within labour markets, with many workers experiencing uncertainty, stress, and apprehension about the continuity of job positions they perceive as threatened (Kalleberg 2009; Aliberti et al. 2018). More recently, the adoption of drastic and restrictive measures to prevent the diffusion of COVID-19 has dramatically impacted labour markets, which are suffering a significant contraction in employment, especially for jobs that imply physical/interpersonal interactions (ILO 2020). Sudden and unexpected transformations in working conditions, also caused by a widespread shift to remote work from home and difficulties in conciliating work with personal life, with increasing efforts to maintain the work-life balance, especially for women, are likely to impact employees’ fear of losing their jobs worldwide. Within this scenario, this paper studies subjective job insecurity in Italy during a period when “COVID-19 has caused structural economic and policy changes that have weakened job security” (Mahmoud et al. 2021).

Italy was the first country in Europe where COVID-19 spread, and since 8 March 2020, when health and safety measures were thought insufficient to cope with the outbreak, the country experienced a lockdown of all nonessential activities with an extraordinary sacrifice to work and businesses. Indeed, Italy has been one of the OECD countries that has experienced the largest reduction in hours worked: − 28% in the initial three months of the crisis (OECD 2020), the Italian government prioritized safeguarding the employment level and workers’ and households’ incomes. With this aim, the government introduced several social security measures and implemented a ban on mass redundancies and individual dismissals for economic reasons from 17 March 2020 and extended this progressively until the end of August 2021. However, many temporary, casual, and seasonal workers, dependent contractors and informal workers lost their jobs. The Italian Statistical Institute (ISTAT 2020) certified a decrease in employment by 3.6% and by 21.6% for temporary workers in the 2020 second trimester. Freedom of movement was allowed since 4 June 2020, with a partial release of the lockdown measures; nevertheless, the second and third waves of the virus made several restrictions still necessary. This scenario of economic uncertainty was likely to influence Italian workers’ expectations about the possibility of continuing to work, increasing the subjective probability of losing their jobs. This was especially true for workers with a regular work contract who, during the pandemic, were protected by the temporary layoff ban upon receiving benefits from the government.

This study focuses on job insecurity as a subjective experience/perception (Lee et al. 2018). It analyses “job tenure insecurity”, which is the fear that employees have of losing their jobs (Gallie et al. 2017).

This article contributes to the literature on subjective job insecurity with findings on Italy during the pandemic, starting with the idea that COVID-19 is a relevant event for employees’ work. Moreover, it investigates several key factors that could be associated with workers’ subjective job insecurity, such as changes in household economic conditions, Italian government social security measures, and remote work. The study employs data drawn from the “Extraordinary Survey on Italian Families in 2020” released by the Bank of Italy in 2021. The literature assesses subjective job insecurity either as a dummy variable or as an ordinal variable and, as a consequence, employs logit/probit models and ordered logit/probit models. The article uses as a dependent variable the responses expressed on a 100-point scale to the question “What is the probability that you will lose your job over the next 12 months?”. Therefore, the dependent variable cannot be considered either a single dummy or an ordinal variable, but a proportional variable that ranges between zero and one. For this reason, we estimate a zero one inflated beta regression model (ZOIB). This method considers both the character of the dependent variable censored in the interval [0, 1] by using a beta distribution function and the accumulation of probabilities in the extreme values of the distribution (zero and one) of a different nature from the intermediate proportions of the distribution (zero and one inflated beta functions). The method assesses all values within an interval of [0, 1]. Therefore, the ZOIB method provides unbiased estimates; however, it shows results on three specifications (zero, one and beta regression), which are not easy to interpret. The main results show that an increase in subjective job insecurity is associated with (1) worsening household economic conditions, (2) receiving a wage guarantee fund in 2019, and (3) increasing the number of people working from home within the household. Moreover, as is well established in the literature, subjective job insecurity during the COVID-19 pandemic is positively associated with having a temporary contract and working in the private sector.

The rest of the paper is structured as follows. The next sections discuss the related literature on subjective job insecurity and describe the Italian economy during the COVID-19 pandemic, providing statements, hypotheses and test of consistency of our dataset. The data and methods employed for the empirical analysis are presented in Sect. 4. Section 5 sets out the findings, while Sect. 6 discusses the main results. Finally, Sect. 7 concludes.

## Related Literature

Subjective job insecurity refers to the perception of a potential threat to continuity in one’s current job; it is subjective because it reflects the degree to which employees consider their jobs to be threatened (Heaney et al. 1994; Shoss 2017). Studying subjective job insecurity is important since the perception of insecurity impacts workers’ mental and physical health, their families, and firms, implying costs for society (Green 2020).

“Job insecurity can be one of the more important stressors in employment situations” (Hartley et al. 1991, p. 84). The perceived threat of unemployment could be as harmful as unemployment itself (Dekker and Schaufeli 1995). Indeed, according to research on stress, the expectation of a stressful condition can be as stressful as the condition itself or even more stressful (Lazarus and Folkman 1984). Because of its psychological impact, job insecurity is assumed to have a negative impact on individual wellbeing (Hellgren et al. 1999), with adverse consequences on health (Holt 1992; De Witte et al. 2015; Caroli and Godard 2016; Cottini and Ghinetti 2018) and the possibility of straining marital and family relationships (Westman et al. 2001). Severe and long periods of stress may reduce job satisfaction (Hellgren et al. 1999), which in turn affects several behaviours among workers, including turnover (Tarigan and Ariani 2015), absenteeism (Schaumberg and Flynn 2017), and effort (Ajzen 2011). Indeed, the literature shows that job insecurity has a detrimental impact on workers’ performance, induces turnover intentions, and causes absenteeism (Staufenbiel and König 2010). These negative outcomes come from a change in workers’ attitudes towards their jobs and a reduction in workers’ commitment to their organizations and job involvement (Ashford et al. 1989), with a potential adverse impact on companies that may be denied effective functioning. In addition, valuable workers, upon experiencing the perception of losing their job, start looking for more stable occupations, which may imply a lack of competent workers for organizations. Furthermore, companies with bad reputations surrounding job security are likely to face difficulties in hiring new employees. Negative outcomes both for workers and organizations turn into potential costs for society at large (Adekiya 2015). Since job insecurity is associated with financial insecurity, high levels of job insecurity may cause drops in consumer spending (Benito 2006), with negative outcomes for economies in terms of a reduction in production and increases in unemployment and poverty. In addition, the adverse health effects of job insecurity impact health care systems, with potential increases in public care expenditures (Pfeffer 1997).

Job insecurity is a perceived phenomenon; however, such a perception generally has its origins in objective threats (Muňoz de Bustillo and de Pedraza 2010; Shoss 2017). Objective threats to job security may be identified in job-related characteristics, organizational features, work organizations, and workplace dimensions (Keim et al. 2014). However, individual variables, including demographic and socioeconomic variables, are also important. Men show a stronger relation between the stress of insecure employment and its negative outcomes than women (Näswall and De Witte 2003), although scholars have not reached a consensus on the role of gender in explaining job insecurity (Shoss 2017; Coron and Schmidt 2021). According to the literature (Naswall and De Witte 2003), older employees face higher levels of job insecurity than younger employees. Research shows that employees with more education report less job insecurity than employees with less education (Kiem et al. 2014). Both income and family structure also influence job insecurity. It is likely that employees with higher incomes have more savings, which is likely to lessen the short-term impact of unemployment, reducing, in turn, perceived job insecurity (Muňoz de Bustillo and de Pedraza 2010). Regarding family structures, because of their parental responsibilities, workers with children are likely to respond more sensitively to a threat to their occupation than workers without children (Näswall and De Witte 2003).

Regarding job-related characteristics, according to the literature (Gallie et al. 2017), temporary workers feel more insecure about their jobs than regular employees. However, temporary jobs could be considered less difficult to regain because temporary workers may have a higher probability (propensity) of regaining employment than permanent workers through a shorter period of unemployment. Public sector workers are less worried about their employment than private sector workers since the public sector is less likely to be affected by cyclical market fluctuations (Chung and Mau 2014; Lübke and Erlinghagen 2014).

## The COVID-19 Pandemic and Job Insecurity in Italy

During the 2020 COVID-19 pandemic, Italian GDP registered its heaviest decline since the Second World War (− 8.9%). The impact on the labour market was significant, but existing social safety nets and extraordinary measures introduced during the emergency helped mitigate it substantially. The sharp drop in hours worked was matched by a much more moderate reduction in the number of employees. The loss of jobs was not homogeneous among the categories of workers; fixed-term and self-employed occupations decreased significantly. The reduction in disposable income caused by the pandemic was extensive and extremely heterogeneous among families. The crisis mainly affected the most vulnerable population, increasing the risk of a rise in inequality in subsequent years. The pandemic also accelerated the digital transformation of the production system; it considerably increased the use of remote work and of new digital technologies. However, the spread of vaccinations and the marked improvement in the global economic environment reinforced expectations of a robust recovery in the second half of 2021 (Bank of Italy 2021).

Within the socioeconomic scenario produced in Italy by COVID-19, we aim to understand the role of the following factors in influencing workers’ subjective job insecurity: household economic conditions, Italian government social security measures, and remote work.

We develop the following statements, hypotheses and test of consistency of our dataset.

### Household Economic Conditions

Household economic conditions are important. A difficult household economic situation heightens perceived job insecurity since the potential loss of a job becomes a threat to the family’s livelihood (Erlinghagen 2008). Furthermore, individuals who live with someone may benefit from the economic support provided by cohabitants (family), with buffers against the experience of job insecurity in times when there is a perceived threat to continued employment (Lim 1996; Anderson and Pontusson 2007). Therefore, the household economic situation will affect how much job insecurity a worker experiences (Chung and Mau 2014). Hence, we test *C*_*1*_ as the consistency of the survey data:

*C*_*1*_ *Household economic difficulties are positively correlated with workers’ subjective job insecurity.*

### Italian Government Social Security Measures

Italian legislation contemplates “Cassa Integrazione Guadagni Ordinaria” (wage guarantee fund, WGF) as provided by the *Italian National Institute of Social Security* (INPS), which consists of a monetary benefit. It is a social shock absorber that supplements or replaces the remuneration of employees who are in precarious economic conditions due to the suspension or reduction of their working hours. The fund is generally reserved for the industrial sector and for companies employing at least 15 employees. During the pandemic, the government extended access to the WGF, as employers had the opportunity to suspend or decrease work activity for events correlated with COVID-19 and to apply for this benefit for reasons related to the COVID-19 emergency. Employees were eligible for a monthly amount of 80% of their salaries. In addition, to avoid delays in payment, companies with fewer than five employees could apply directly to the INPS. For those not covered by the WGF, because they were not employees or not employed at the time, such as self-employed and seasonal workers, there was a special allowance of €600 a month for March and April 2020, which increased to €1000 for May 2020.

Social safety nets allow workers to protect employment and integrate their incomes, so welfare remains stable for their duration. Thus, on the one hand, the WGF could help reduce the fear of job loss, keeping welfare and employment unchanged. On the other hand, the WGF could increase workers’ perceived probability of losing their jobs because such a policy does not provide workers with long-term guarantees. From the above considerations, we posit as *S*_*1*_ the following statement:

*S*_*1*_ *The net effect of the WGF on subjective job insecurity is an empirical issue.*

### Bonus for Babysitting Services

In Italy, public services for children and formal childcare are very limited. Grandparents support parents’ participation, especially mothers’ participation, in the paid labour market, playing an important role in informal childcare. However, during the lockdown, grandparents were not allowed to meet children because of social distancing prescriptions. With schools closed for several months, mothers bore most of the burden, and when possible, they asked to work from home; alternatively, parents decided to work fewer hours, and in some extreme cases, they decided not to work at all for childcare reasons. To support working parents in 2020, the Italian government introduced a bonus for babysitting services. It was introduced first by the “Cure Italy Decree”, and it is still available for working parents. The bonus includes a voucher for babysitting services, and its amount was set to up to a maximum of €600 for services provided in 2020, starting on 5 March. The “Relaunch Decree” increased the voucher from €600 to €1200 (it could be used over two months), and it could also be used for enrolment in summer camps.

It is likely that this measure of financial support decreases parents’ fears of losing their jobs (especially for mothers) for parents who find it difficult to conciliate working time and childcare. The availability of extra funds to pay for babysitting services offers an opportunity to arrange for childcare without renouncing work. Hence, we posit as *H*_*1*_ the following hypothesis:

*H*_*1*_ *The bonus for babysitting services is negatively associated with the fear of job loss because it allows workers to conciliate working time and childcare.*

### Remote Work

Working from home could provide workers with some advantages and potential disadvantages.

First, it represents an important change in working conditions for workers who generally do not work from home. During the pandemic, this change happened very quickly, and it was not expected. This implied several benefits and difficulties for workers who generally worked in a standard work environment. Regarding benefits, working from home saves time and costs spent commuting and, in some cases, job schedule flexibility. On the other hand, working from home involves social isolation, which means a lack of opportunity to share ideas, concerns, and solutions through face-to-face relationships. This, in turn, implies a reduction or even a total absence of support from colleagues and bosses. The weakening of a sense of belonging to a network represents another drawback of working from home. These disadvantages, in addition to the struggle to balance time for private life and time for work life, which could overlap, may produce feelings of uncertainty for workers and therefore job insecurity.

From the above considerations, we posit as *S*_*2*_ the following statement:

*S*_*2*_ *The net effect of remote work on subjective job insecurity is an empirical issue.*

## Data and Methods

To study Italian workers’ subjective job insecurity, we employ data drawn from the “Extraordinary Survey on Italian Families in 2020” carried out in 2020 and released by the Bank of Italy in 2021.^1^ Data were accessed and downloaded via the Bank of Italy server. The survey offers an account of Italy during the COVID-19 pandemic and provides interesting information on the economic conditions and expectations of households as well as on Italian government social security measures. The interviews were conducted (between the end of August and the beginning of September) via a remote connection device (dialogue) and involved more than 2346 households, and 881 of these also participated in the last April edition. Indeed, the survey is a second wave of a survey conducted in April 2020, released in December 2020. However, the first wave is strongly limited in terms of questions and topics, while the second having a broader scope. For our analysis, we used only the last wave as a cross section because it provides information about job insecurity. The survey provides weights that were designed on a stratified sample, which is based on age, gender and five macro level regions. We analyse a subsample of people of working age, excluding retired and unemployed individuals and students. Upon eliminating all missing values for dependent and independent variables, our sample includes 1025 observations.

### Methodology

The estimations of the marginal effects of the independent variables on the perceived probability of job loss consider the characteristics of our dependent variable. In the literature, subjective job insecurity is assessed either as a dummy variable or as an ordinal variable. As a consequence, the methodologies adopted are logit/probit models and ordered logit/probit models (Erlinghagen 2008; Muňoz de Bustillo and de Pedraza 2010; Gallie et al. 2017).

### Dependent Variable

We selected *Jobless* as our dependent variable*,* i.e., responses to Question n. 13 of the survey: “What is the probability that you will lose your job over the next 12 months?”.

Responses are expressed on a 100-point scale, with a “0” value denoting that the respondent believes that he or she will not lose his or her job and a “100” value denoting that the respondent believes that he or she will lose his or her job. For two reasons, our dependent variable requires a methodology of estimation that differs from those applied by the literature on perceived job insecurity.

First, we have a variable that cannot be considered either a single dummy or an ordinal one. We use continuous values from 0 to 100 that we scale between 0 and 1. When we exclude the two extremes from the analysis, we obtain a fractional variable that is usually estimated with a fractional model if we can consider the variable continuous. In this case, using an OLS model produces biased estimates with values larger than one and less than zero. Among the models used to estimate fractional variables is the beta regression model. It is a very flexible method that allows us to consider a wide range of distributions between ]0, 1[. Therefore, it may be employed even when there are many observations that are close the extreme points (Ferrari and Cribari-Neto 2004).

Second, we must consider the probability that the zero event occurs (if the respondent believes that he or she will not lose his or her job) and the probability that the one event occurs (if the respondent believes that he or she will lose his or her job). Since boundaries of zero and one are included in the dependent variable, not estimating them is likely to generate biased results if there are a large number of observations. In this case, a zero/one inflated beta regression is assessed (Cook et al. 2008; Ospina and Ferrari 2010, 2012; Buis 2020).^2^ The zero/one inflated regression is estimated as a logit model. In the one inflated regression, the dependent variable is equal to 1 if the probability of losing one’s job is equal to 1 (the respondent believes that he or she will lose his or her job) and zero otherwise. In the zero inflated regression, the dependent variable is equal to 1 if the probability of losing one’s job is equal to 0 (the respondent believes that he or she will not lose his or her job) and zero otherwise. Hence, in this last setting, marginal effects have the opposite sign and meaning than the probability of losing a job when it takes values within the range of [0, 1]. ZOIB estimates include a beta regression equation and two logit equations for the two extreme points in the interval [0, 1].

**Fig. 1:**
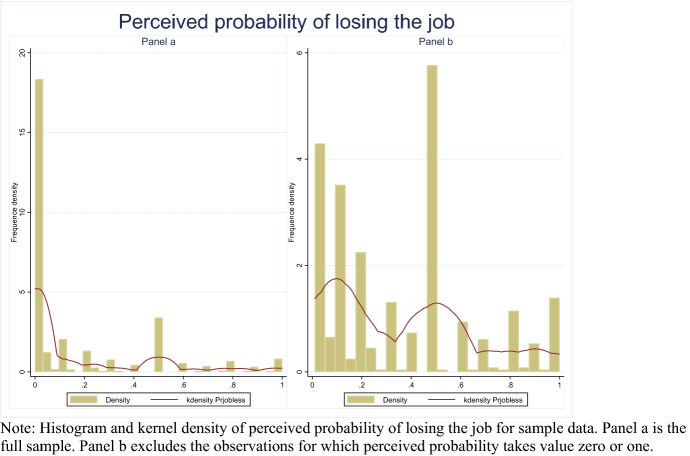
Distribution of perceived probability of losing the job. Histogram and kernel density of perceived probability of losing the job for sample data. **a** The full sample. **b** Excludes the observations for which perceived probability takes value zero or one

The distribution of the dependent variable is reported in Fig. 1. Panel (a) shows the distribution including boundaries, while Panel (b) excludes boundaries. The descriptive statistics of *Jobless* are reported in Table 1. Descriptive statistics for each subgroup of *Jobless* are described in Table 4 in Appendix A.

**Table 1:** Descriptive statistics

Variable	Label	Obs	Mean	Std. dev.	Min	Max
Jobless	Perceived probability of losing the job	1025	0.156	0.263	0	1
*Short-term expectations on the future of the Italian economy*						
Opinion	PCA predicting the worker opinion about the path of the economy and of the job market for the next 12 months	1025	− 0.018	1.335	− 3.016	1.724
*Variables related to household economic conditions*						
RedIncome	= 1 if during the pandemic there has been a reduction in the household income	1025	0.345	0.476	0	1
Make-ends-meet	= 1 if before the pandemic, considering the household’s total monthly income, the household has difficulties to make ends meet	1025	0.525	0.5	0	1
LHMIncome	Log of household monthly net income	1025	7.427	0.632	5.011	9.21
*Variables related to Government interventions*						
WGF19	= 1 if the worker has received a wage guarantee fund in 2019	1025	0.145	0.353	0	1
WGF20	= 1 if the worker has received a wage guarantee fund in 2020	1025	0.216	0.411	0	1
Support20	= 1 if the worker has received funds for independent workers in 2020	1025	0.15	0.357	0	1
Bonusbb20	= 1 if the worker has received a babysitter bonus in 2020	1025	0.069	0.254	0	1
*Remote work*						
Remotework	= 1 if at least a member of the household has worked from home at least three days a week during the lockdown	1025	0.473	0.5	0	1
Remote	Number of remote workers/number of household components	1025	0.27	0.355	0	1
*Variables related to job characteristics*						
Self-employed	= 1 if the worker is self-employed	1025	0.185	0.389	0	1
Temporary	= 1 if the worker has a temporary job	1025	0.078	0.268	0	1
Private	= 1 if the worker is a private sector worker	1025	0.774	0.419	0	1
Agriculture	= 1 if the worker is agricultural sector worker	1025	0.034	0.182	0	1
Manufacture	= 1 if the worker is a manufacturing sector worker	1025	0.218	0.413	0	1
RedEmploy1	= 1 if during the lockdown one component of the household lost the job	1025	0.74	0.439	0	1
RedEmploy2	= 1 if during the lockdown more than one component of the household lost the job	1025	0.084	0.277	0	1
*Variables related to individual and household characteristics, place of residence*						
Female	= 1 if the worker is female	1025	0.235	0.424	0	1
Age	Worker age	1025	49.351	10.751	23	85
Low	= 1 if the worker has a lower education	1025	0.353	0.478	0	1
Diploma	= 1 if the worker has a higher education	1025	0.386	0.487	0	1
Graduate	= 1 if the worker is graduated and has an education higher than graduated	1025	0.222	0.416	0	1
Ncomp	Number of household components	1025	2.895	1.277	1	7
Nolookchildren	= 1 if during the schools’ closure components of the family kept working although had children aged less than 14 years to look after	1025	0.919	0.273	0	1
Morechores	= 1 if during the lockdown the worker participated more intensively to household chores	1025	0.221	0.415	0	1
Lesschores	= 1 if during the lockdown the worker participated less intensively to household chores	1025	0.085	0.279	0	1
POP1	= 1 if the worker’s municipality counts less than 5000 persons	1025	0.134	0.34	0	1
POP2	= 1 if the worker’s municipality counts between 5000 and 10,000 persons	1025	0.151	0.358	0	1
POP3	= 1if the worker’s municipality counts between 10,000 and 30,000 persons	1025	0.266	0.442	0	1
POP4	= 1 if the worker’s municipality counts between 30,000 and 100,000 persons	1025	0.202	0.402	0	1
POP5	= 1 the worker’s municipality counts more than 100,000 persons	1025	0.247	0.431	0	1
North	= 1 if the worker lives in the Northern macro area	1025	0.51	0.5	0	1
Center	= 1 if the worker lives in the Central macro area	1025	0.224	0.417	0	1
Mezzogiorno	= 1 if the worker lives in the Southern macro area	1025	0.265	0.442	0	1
*Variables related to Covid-19 pandemic*						
Ldeaths	Log of the number of deaths in (Aug–Sep) 2020 compared to the average deaths of (Aug–Sep) 2015–2019 (ISTAT)	1025	5.429	0.46	4.104	6.045
VarConUnempl	Unemployment conjunctural variation (ISTAT)	1025	11.398	9.233	− 0.83	50.498

Descriptive statistics are limited to the regression sample

### Model Specification and Independent Variables

Taking into consideration that we have three different equations, two logit and a beta regression, each of them has the same variable specification described in the following formula:

1 $$ \begin{aligned} Jobless & = f(\alpha + \beta \, Opinion + Econdition \, \gamma + Policies \, \delta + \theta \, Remotework \\ & \quad + Job \, features \, \upsilon + HHTraits \, \rho + Covid19 \, \psi + \mu ) \\ \end{aligned} $$

Unobservable characteristics of the workers (feature traits such as pessimism or optimism) could influence their perceptions of job insecurity. Hence, to control for the above effect, we introduce the variable *Opinion*. This variable is obtained by a principal component analysis (PCA) of two questions of the survey asking the worker to predict the path of the economy in general and of the job market for the next twelve months. For both questions, answers are ordered from the most optimistic to the most pessimistic predictions and are highly correlated to each other. Therefore, the resultant variable (*Opinion)* ranges from the most optimistic to the most pessimistic values and can be considered a continuous variable.

*Econdition* is a set of three variables that tests C_1_ regarding the household economic conditions specified in Sect. 3. The first is *Redincome*, a binary variable that indicates whether there was a reduction in household income during the pandemic*.* The second dummy is *Make-ends-meet*, which indicates whether, before the pandemic, considering the household’s total monthly income, a household had difficulties paying expenses. The third is the logarithm of household monthly net income *LHMIncome*. The survey provides 23 nonhomogeneous income classes. We build a continuous variable by taking the average values of each class.

*Policies* is a set of four dummy variables that tests *S*_*1*_ and *H*_*1*_. The variables indicate if a worker’s household received a wage guarantee fund in 2019 (*WGF19*) or 2020 (*WGF20*), funds for independent workers in 2020 (*Support20*), or a babysitter bonus in 2020 (*Bonusbb20*).

*Remotework* is a variable that tests *S*_*2*_ related to the effect of remote work. It is a dummy variable that indicates whether at least one member of a household works from home at least three days a week during the lockdown. We also replaced *Remotework* with *Remote* (see Table 3), which is the number of people in remote work divided by the number of household components that is a proportional variable that accounts for the intensity of the phenomenon. We do not control for both variables at the same time because they are highly correlated to each other.

We consider a set of variables related to job features. We know if a worker is self-employed or an employee, has a temporary or indefinite contract, and works in the private or public sector. Thus, we build the following dummy variables: *Self-employed*, *Temporary* and *Private*. *Agriculture* and *Manufacture* are two dummy variables that indicate the macrolevel sector within which the interviewee works. We also consider the variables *RedEmploy1* and *RedEmploy2,* which indicate if during the lockdown one component of a household experienced job loss and if during the lockdown more than one component of the household experienced job loss.

We also control for individual and demographic characteristics (*HHtraits*). Regarding gender, we consider *Male* as the reference group. We consider a worker’s age as a discrete variable, *Age*. We include three dummies for education: *Graduate* (workers with a graduate education and with a postgraduate education), *Diploma* (workers with higher education)*,* and *Low* (workers with lower education). We consider a discrete variable *Ncomp* as the number of household components.

We control for *Nolookchildren*, a dummy variable that indicates if during the school closures, components of the family kept working despite the presence of children aged less than 14 years to look after. We consider two variables regarding the distribution of household chores: *Morechores* if during the lockdown there was more intensive participation in household chores and *Lesschores* for less intensive participation. These two variables do not cover all possible results because there may have been no changes in the distribution.

Considering the substantial heterogeneity among the Italian regions, we consider three macrolevel aggregations of regions (*North*, *Centre,* and *Mezzogiorno*). We also consider whether interviewees lived in very small, small, medium-sized, large, or very large towns (*POP1*, *POP2*, *POP*3, *POP*4, and *POP*5, respectively).

Finally, to capture effects of the COVID-19 pandemic on subjective job insecurity, we added two variables to our regressions, both obtained from the Italian Statistical Institute. The first variable considers the number of deaths due to COVID-19 (*Ldeaths*), measured as the logarithm of the number of deaths in August and September of 2020 in excess with respect to the average of 2015–2019 for municipalities of different population classes (less than 5000, between 5000 and 10,000, between 10,000 and 30,000, between 30,000 and 100,000 and more than 100,000) for five macro regions (North-East, North-West, Centre, South and Islands). The second (*VarConUnempl*) is the conjunctural variation of the rate of unemployment between the second (Apr-Jun) and third trimesters (Jul-Sep) of 2020 for four categories of education (Elementary, Low, Diploma and Graduate), for two age classes (less than 35 years and more than 35 years), for females and males, and for four macro regions (North-East, North-West, Centre and South). Table 1 shows the descriptive statistics for our sample.

## Results

Figure 1, panel (b) denotes that the *Jobless* distribution for the proportional part can be considered continuous; panel (a) shows that it is impossible to exclude zero inflation from the estimates. The same conclusions are drawn from the descriptive statistics in Table 4, where we see that less than 50% of the observations are included in the subsample for which *0* < *Jobless* < *1* and more than 50% of the observations are included in the subsample for which *Jobless* = *0*. A small number of observations is included in the subsample for which *Jobless* = *1*.

The restricted number of observations could suggest excluding the one inflation beta regression. However, we estimate the beta regression, the zero inflated regression, and the one inflated regression and examine the last estimations with caution.

We report ZOIB average marginal effects in Table 2. The first column shows average marginal effects without distinguishing between the contribution of each of the three equations that constitute the ZOIB model. The second column illustrates the results for the one inflated regression regarding workers who feel sure to lose their jobs. The third column shows the average marginal effects of the beta regression regarding workers who present a positive probability of losing their jobs; and the last column reports average marginal effects of the zero inflated regression, concerning workers who are sure to not lose the job.

**Table 2:** Average marginal effects of zero and one inflation beta regression for subjective probability of losing the job

	(1)	(2)	(3)	(4)
	ZOIB	One Inflation	Beta	Zero Inflation
	b/se	b/se	b/se	b/se
Opinion	0.019** (0.007)	0.009 (0.007)	0.011 (0.008)	− 0.021^+^ (0.012)
RedIncome	0.083*** (0.021)	0.006 (0.019)	0.058** (0.023)	− 0.168*** (0.034)
Make-ends-meet	0.025 (0.025)	− 0.029 (0.023)	0.063** (0.024)	− 0.071* (0.034)
LHMIncome	− 0.002 (0.023)	0.020 (0.022)	− 0.049* (0.024)	− 0.009 (0.029)
WGF19	0.012 (0.040)	0.005 (0.038)	0.064^+^ (0.033)	0.068 (0.053)
WGF20	− 0.014 (0.035)	− 0.019 (0.034)	− 0.044 (0.029)	− 0.074 (0.048)
Support20	0.041 (0.035)	0.029 (0.031)	− 0.006 (0.042)	− 0.061 (0.053)
Bonusbb20	− 0.611*** (0.160)	− 0.744*** (0.176)	0.002 (0.040)	− 0.075 (0.064)
Remotework	0.042^+^ (0.024)	0.027 (0.023)	− 0.023 (0.024)	− 0.097** (0.035)
Ldeaths	0.021 (0.029)	− 0.033 (0.027)	0.064* (0.031)	− 0.069 (0.044)
VarConUnempl	0.001 (0.001)	0.001 (0.001)	0.000 (0.002)	− 0.002 (0.002)
Job features	Yes	Yes	Yes	Yes
Individual and household features	Yes	Yes	Yes	Yes
City size	Yes	Yes	Yes	Yes
Macro region	Yes	Yes	Yes	Yes
*N*	1025	20	438	567

The first column shows average marginal effects (AME) for the entire model; the second column shows AME for the perceived probability of losing the job equal to one (Logit); the third column shows AME for proportion ranging between zero and one (0,1) estimated through a beta regression; the fourth column shows AME for the perceived probability of losing the job equal to zero (Logit). In the last column, perceived probability of losing the job has an opposite sign because the logit model takes value 1 as the probability is equal to zero. Robust standard error in parentheses account for the survey weights, p-value: *** p < 0.001, ** p < 0.01, * p < *0.5,* ^+^ *p* < *0.1*

Column 1 shows that *Opinion* is positively correlated with *Jobless* and significant at the 1% level. Increasingly negative expectations about the path of the economy and of the job market for the next 12 months is associated with an increase in the subjective probability of job loss by 1.9% points. As *Opinion* ranges from − 3 to 1.7 (Table 1), the effect on *Jobless* varies from the lowest (− 5.7% points) to the maximum level (3.3% points), with a maximum difference of 9% points. For the zero inflation equation (last column), the marginal effect is significant at the 10% level and higher (2.1% points).

From the variables that test *C*_*1*_, *Redincome* shows a positive, significant at the 1% level, correlation with *Jobless* (ZOIB). A reduction in household income during the pandemic is associated with an increase in the subjective probability of job loss by 8.3% points. In Column 3, the marginal effect of *Redincome* decreases slightly (5.8% points) but remains significant (1% level). In the zero inflated estimates (last column), the marginal effect is significant at 1% level and broader (16.8% points). Concerning *Make-ends-meet,* the marginal effect is positive but not significant in the ZOIB. Nevertheless, it is significant in the beta and zero inflated regressions. In Column 3, *Make-ends-meet* is significant at the 1% level, and its marginal effect is equal to 6.3% points. In Column 4, *Make-ends-meet* is significant at the 5% level with a marginal effect equal to 7.1% points. Finally, the marginal effect of *LHMIncome* is negatively significant at the 5% level only in the beta regression model (Column 3). Increasing household monthly net income is associated with a decrease in the perceived probability of losing one’s job by 4.9% points. As *LHMIncome* ranges from 5 to 9 (Table 1), the effect on *Jobless* varies from the lowest (− 25% points) to the maximum level (− 45% points), with a maximum difference of 20% points.

For *S*_*1*,_ our variables of interest are *WGF19*, *WGF20* and *Support20*. Our results show that the WGF19 is positively significant at 10% only in the beta regression model (Column 3). Receiving a redundancy fund in 2019 is associated with an increase in the probability of job loss by 6.4% points. The marginal effects of *WGF20* and *Support20* are never significant.

To test *H*_*1*_, we consider *Bonusbb20.* In the ZOIB specification*, Bonusbb20* is negatively correlated with *Jobless* and statistically significant at 1% level. When a household received a babysitter bonus for children in 2020, the perceived probability of job loss is associated with a decrease of 61.1% points. However, the results are not significant in the beta and zero estimations (Columns 3 and 4). This means that the results are driven by the one inflation specification. Indeed, in Column 2, the marginal effect of *Bonusbb20* is equal to 74.4% points. Since the number of observations in the beta regression specification is very small, the results shown in Column 1 should be interpreted with caution.

Concerning *S*_*2*_, our variable of interest is *Remotework.* In Column 1*, Remotework* is positively correlated with *Jobless* and statistically significant at 10% level. Increasing the number of people working from home within a household is associated with an increase in the perceived probability of job loss of 4.2% points. The marginal effects of *Remotework* are not significant in Columns 2 and 3 but are significant in Column 4 at 1% level. Indeed, in zero inflated estimates, increasing the number of people working from home within the household is associated with an increase in *Jobless* of 9.7% points.

For variables related to the COVID-19 pandemic, Table 2 shows that the marginal effect of *Ldeaths* is statistically significant (5% level) and positive only in Column 3. Increasing the number of deaths (the logarithm) in 2020 compared to the average deaths of 2015–2019 is associated with an increase in the perceived probability of job loss of 6.4% points. As *Ldeaths* ranges from 4.1 to 6 (Table 1), the effect on *Jobless* varies from the lowest (26% points) to the maximum level (38% points), with a maximum difference of 12% points. The marginal effects of *VarConUnempl* are never significant.

In the analysis of *Jobless*, the variables related to job characteristics, individual and household characteristics, and the place of residence are important as well (Böckerman 2004; Erlinghagen 2008; Muňoz de Bustillo and de Pedraza 2010; Bernhardt and Krause 2014). We only comment on results that are statistically significant.^3^ Regarding gender, the marginal effect is statistically significant at 10% level and positive only in the one inflated specification. Being female is associated with an increase in *Jobless* of 5.6% points. In addition, only in the one inflated specification the marginal effects of *Age* (0.2% points for each additional year) are significant at the 10% level and negative only in the one inflated specification. Moreover, according to the literature (Anderson and Pontusson 2007; Chung and Mau 2014; Gallie et al. 2017; Lowe 2018), being employed with a temporary contract is significant and associated with an increase in *Jobless* in all specifications. Being employed in the private sector is always significant but not in the one inflated specification. The marginal effect (6.4% points) of *RedEmploy1* is statistically significant at 1% level and negative in the one inflated estimation.

The key difference between Tables 2 and 3 is that we substitute the *Remotework* variable with the *Remote* variable.^4^ Table 3 shows that the overall results remain unchanged.

**Table 3:** Average marginal effects of zero and one inflation beta regression for subjective probability of losing the job

	(1)	(2)	(3)	(4)
	ZOIB	One inflation	Beta	Zero inflation
	b/se	b/se	b/se	b/se
Opinion	0.019** (0.007)	0.009 (0.006)	0.011 (0.008)	− 0.021^+^ (0.012)
RedIncome	0.084*** (0.021)	0.009 (0.019)	0.056* (0.022)	− 0.167*** (0.034)
Make-ends-meet	0.027 (0.025)	− 0.027 (0.023)	0.063** (0.024)	− 0.071* (0.034)
LHMIncome	0.000 (0.023)	0.020 (0.022)	− 0.047* (0.023)	− 0.014 (0.029)
WGF19	0.014 (0.040)	0.007 (0.039)	0.065* (0.033)	0.070 (0.053)
WGF20	− 0.012 (0.035)	− 0.018 (0.035)	− 0.046 (0.029)	− 0.079 (0.048)
Support20	0.041 (0.035)	0.029 (0.031)	− 0.004 (0.042)	− 0.060 (0.053)
Bonusbb20	− 0.629*** (0.169)	− 0.764*** (0.183)	0.003 (0.040)	− 0.080 (0.064)
Remote	0.047 (0.035)	0.046 (0.032)	− 0.054 (0.036)	− 0.106* (0.050)
Ldeaths	0.021 (0.029)	− 0.032 (0.027)	0.062* (0.031)	− 0.069 (0.044)
VarConUnempl	0.001 (0.001)	0.000 (0.001)	0.000 (0.002)	− 0.002 (0.002)
Job features	Yes	Yes	Yes	Yes
Individual and household features	Yes	Yes	Yes	Yes
City size	Yes	Yes	Yes	Yes
Macro region	Yes	Yes	Yes	Yes
*N*	1025	20	438	567

The first column shows average marginal effects (AME) for the entire model; the second column shows AME for the perceived probability of losing the job equal to one (Logit); the third column shows AME for proportion ranging between zero and one (0,1) estimated through a beta regression; the fourth column shows AME for the perceived probability of losing the job equal to zero (Logit). In the last column, perceived probability of losing the job has an opposite sign because the logit model takes value 1 as the probability is equal to zero. Robust standard error in parentheses account for the survey weights, p-value: ***p < 0.001, **p < 0.01, *p < *0.5,* ^+^*p* < *0.1*

Since the one inflated specification includes a very small number of observations, we estimate Eq. (1) without considering the subgroup for which *Jobless* = 1. Tables 5 and 6 in Appendix A report the results. New estimations show that the results remain substantially unaffected. However, in the specifications, the marginal effect of the *Bonusbb20* variable is not significant.

## Discussion

In the discussion of the results, we focus primarily on the beta and the zero inflated regressions (and when it could be helpful on the ZOIB and one inflated model). This way of proceeding is determined by the fact that in the one inflated regression, the sample is quite small, and for this reason, the results must be interpreted with caution.

From the sign, size and significance of the marginal effects of the variables related to household economic conditions, i.e., *Redincome* and *Make-ends-meet,* we validate C_1_: worsening household economic conditions is associated with workers’ greater subjective job insecurity. These findings are in line with previous studies (Anderson and Pontusson 2007; Chung and Mau 2014; Ellonen and Nätti 2015) and indicate that a difficult household economic situation enhances workers’ subjective job insecurity since the potential loss of a job threatens the family’s living standards. Additionally, the negative marginal effect of *LHMIncome* shows that benefiting from economic support provided by cohabitants (family) buffers against subjective job insecurity when people believe their continued employment to be threatened, which occurred during the COVID-19 pandemic.

Several macroeconomic and microeconomic studies find that during economic crises, short-term work (STW) policies have a positive effect in keeping workers employed (Boeri and Bruecker 2011; Cahuc et al. 2018; Giupponi and Landais 2020; Gehrke and Hochmuth 2021; Kopp and Siegenthaler 2021). Among others, Cahuc et al. (2018) and Kopp and Siegenthaler (2021), using French and Swiss data (microeconomic data), show that STW schemes prevent dismissal.

In interpreting this evidence to explain our results, we do not find that the wage guarantee fund buffers against subjective job insecurity. First, from ZOIB results, if the WGF does not have an association with subjective job insecurity, this may indicate that such temporary policy may have a positive impact in terms of keeping welfare and employment unchanged but this positive effect is countered by the fact that it is a short-term policy. Second, in the beta regression estimates, the positive correlation between WGF and the perceived probability of job loss may point out that the positive impact provided by the WGF in terms of keeping welfare and employment unchanged is dominated by the fear of being fired when the short-term guarantees offered by WGF end.

Giupponi and Landais (2020) help us explain the above results. The authors show that the Italian STW scheme postpones rather than prevents dismissals. Using unique Italian administrative social security data from Italy for the Great Recession and quasi-experimental variation in STW policy rules, the authors find large and positive effects of the WFG on headcount employment. In the case of persistent shocks, the authors find that the long-run effects of STW were null for low-productivity firms. The employment and earnings of workers from low-productivity firms treated by STW were the same as those of laid-off workers in similarly low-productivity firms three years after treatment.

Moreover, Arranz et al. (2018), using 2009 Spanish microdata, find that STW schemes were marginally successful in achieving the continuity of jobs within firms. According to the authors, their result approximates the empirical evidence from the microeconomic literature, which in general has not found any significant net effects of STW on employment (Bellmann, et al. 2012; Calavrezo, et al. 2010).

The results for Hypothesis H_1_ are equivocal. From the ZOIB and one inflated regressions, receiving a babysitter bonus for children in 2020 is associated with a decrease in workers’ perceived probability of losing their jobs. Nevertheless, according to the beta and at the zero regressions, the results are not statistically significant. Although interesting, as we note, the *Bonusbb20* findings should be considered carefully, as the sample of the one inflated specification is too small. From the above statement, we observe that during the COVID-19 pandemic, the closure of schools and childcare services as well as restricted access to care through extensive family and friends caused parents to struggle to provide childcare at home. Before the lockdown, grandparents provided most Italian families with childcare. Within this scenario, the Italian government bonus for babysitting has not been able to guarantee to workers a conciliation of working time and childcare. Thus, our results are not in line with the literature according to which subsidized childcare helps work-family balance, since it allows parents to balance their work and family responsibilities (Blair-Loy 2010; Correll et al. 2014).

For *S*_*2*_, the results show that increasing the number of people working from home within a household is associated with an increase in *Jobless* in zero inflated estimates. We considered two proxies for working from home called *Remotework* and *Remote,* whose marginal effects on *Jobless* have the same sign and a similar magnitude.

However, it should be recognized that although we control for macro level activity sectors, *Opinion* and, the number of deaths due to the Covid-19 pandemic, the positive correlation between working from home and perceived job insecurity might be driven by specific activity sectors and occupations (i.e., high skilled) where remote workers are more numerous, by different subjective perceptions of remote workers as well as by the health emergency due to the Covid-19 pandemic.

The results on remote work seem in line with the literature: “remote working during a pandemic in weak labour markets can increase job insecurity” (Ghislieri et al. 2022). This seems to apply in the Italian case. According to Ghislieri et al. (2022), changes in working conditions imposed by generalized lockdown and the widespread diffusion of remote work have made employers and employees feel uncertain about their jobs. This was likely to happen since working from home increased “job demands” with workers expected to be almost always available even outside of working hours. Working from home using technological devices implied in many cases responding to job-related questions with no right to digitally disconnect, which only recently has received attention. Being continuously connected and working beyond formal working hours may have made it difficult to achieve balance with the home/family environment, especially for workers who had to look after children and experiencing interference from other family members, neighbours, and friends. This is likely to make workers worried about their jobs since their performance does not match employers’ expectations. Indeed, perceived job insecurity is a subjective perception; generally, it has its origins in any objective threat that produces uncertainty in workers’ minds. However, people can feel worried about their jobs independent of an existent threat to job loss, in that it involves an anticipation of a stressful event related to the continuation of one’s job (Sverke et al. 2002).

According to another branch of the literature, among other factors, perceived job insecurity is influenced by social support within the workplace (see, among others, Dormann and Zapf 1999; Halbesleben 2010; Wang et al. 2019). Working from home implies social isolation. Being disconnected from colleagues prevents the sharing of ideas and collaboration with coworkers, which are typical of working in teams. This makes workers insecure and worried about their jobs. Furthermore, being away from a standard work environment makes workers feel invisible, which results in worries about opportunities for promotion, incentives, and positive performance evaluations (Cooper and Kurland 2002).

According to the literature, a country’s labour market conditions and economic scenario are predicted to impact individuals’ subjective job insecurity (Anderson and Pontusson 2007; Chung and Van Oorschot 2010; Ellonen and Nätti 2015). Our findings on *Opinion*, *VarConUnempl* and *Ldeaths* seem to be in line with the literature. On the one hand, the results show that pessimistic expectations about the future of the economy and about labour market performance threaten subjective job security (Tables 2 and 3, Column 4). In addition, the results seem to suggest that expectations impact subjective job security rather than government policy. On the other hand, subjective job insecurity is not affected by conjunctural variation of unemployment but by the increasing number of deaths in 2020 compared to the average deaths of 2015–2019. This last result may be linked to perceptions of overall insecurity within the economy, which create stress for workers.

## Conclusion

Using data from the “Extraordinary Survey on Italian Families in 2020” conducted by the Bank of Italy in 2021 and the Zero One Inflated Beta model, this paper investigates Italian workers’ perceived probability of losing their jobs during the COVID-19 pandemic. When Italian GDP registered its heaviest decline since the Second World War, the impact on the labour market was significant, and the Italian government introduced several measures to both support financial workers who could not keep working during the pandemic and protect employees from dismissals.

For this scenario, this paper shows that (1) worsening household economic conditions before and during the lockdown, (2) receiving a wage guarantee fund in 2019, and (3) increasing the number of people working from home within the household are associated with an increase in subjective job insecurity.

Another relevant finding concerns pessimistic expectations of the future of the Italian economy. Having pessimistic expectations about the future of the Italian economy is associated with an increase in subjective job insecurity, which also shows a positive association with having a temporary contract and working in the private sector.

Finally, the results on working from home are very interesting. Although working from home has some advantages in terms of providing flexibility and the freedom to determine when and where to perform job activities and savings on commuting costs and time, our results show a positive correlation between working from home and perceived job insecurity, with the limitations discussed in the previous section. During the pandemic in Italy, working from home burst and it did not work very well. Thus, it seems appropriate not to confuse a “temporary abnormal”, which is helpful when people cannot interact for virus transmission reasons, with a “new normal”, which implies to consider whether working arrangements, as a complement rather than a substitute for in office activities, may improve the resilience of jobs. The main recommendation for policy-makers is, thus, to be cautious and to avoid generalizing work from home practices. Anyway, future research is needed on this point.

Although its findings are interesting, the econometric analysis has the limitations of considering only the Italian context, of using standard omitted variables generally considered in the literature on the topic, and of being cross sectional and for this reason evaluating correlations instead of causation. Despite these limitations, the Bank of Italy dataset has the benefit of being very recent and representative of the Italian population.

## Appendix A

See Tables 4, 5 and 6.

**Table 4:** Descriptive statistics for each sub-group of *Jobless*

Variables	Full		Jobless = 1		0 < Jobless < 1		Jobless = 0	
	Mean	S.D.	Mean	S.D.	Mean	S.D.	Mean	S.D.
Opinion	− 0.018	1.335	0.007	1.152	0.122	1.363	− 0.126	1.31
RedIncome	0.345	0.476	0.5	0.513	0.475	0.5	0.24	0.427
Make-ends-meet	0.525	0.5	0.55	0.51	0.578	0.495	0.483	0.5
LHMIncome	7.427	0.632	7.309	0.902	7.42	0.594	7.437	0.65
WGF19	0.145	0.353	0.1	0.308	0.174	0.379	0.125	0.331
WGF20	0.216	0.411	0.2	0.41	0.279	0.449	0.168	0.374
Support20	0.15	0.357	0.2	0.41	0.196	0.398	0.113	0.317
Bonusbb20	0.069	0.254	0	0	0.082	0.275	0.062	0.241
Remotework	0.473	0.5	0.55	0.51	0.473	0.5	0.471	0.5
Remote	0.27	0.355	0.296	0.36	0.264	0.351	0.274	0.359
Self-employed	0.185	0.389	0.15	0.366	0.224	0.417	0.157	0.364
Temporary	0.078	0.268	0.4	0.503	0.128	0.334	0.028	0.166
Private	0.774	0.419	0.75	0.444	0.92	0.271	0.661	0.474
Agriculture	0.034	0.182	0.1	0.308	0.025	0.157	0.039	0.193
Manufacture	0.218	0.413	0.05	0.224	0.288	0.453	0.169	0.375
RedEmploy1	0.74	0.439	0.6	0.503	0.744	0.437	0.741	0.439
RedEmploy2	0.084	0.277	0.1	0.308	0.084	0.278	0.083	0.276
Female	0.235	0.424	0.3	0.47	0.219	0.414	0.245	0.431
Age	49.351	10.751	46.95	11.985	48.694	10.6	49.944	10.8
Low	0.353	0.478	0.55	0.51	0.361	0.481	0.34	0.474
Diploma	0.386	0.487	0.25	0.444	0.381	0.486	0.395	0.489
Graduate	0.222	0.416	0.15	0.366	0.212	0.409	0.233	0.423
Ncomp	2.895	1.277	3.2	1.196	2.909	1.271	2.873	1.285
Nolookchildren	0.919	0.273	0.9	0.308	0.909	0.288	0.928	0.259
Morechores	0.221	0.415	0.15	0.366	0.237	0.426	0.212	0.409
Lesschores	0.085	0.279	0.05	0.224	0.091	0.288	0.081	0.273
POP1	0.134	0.34	0.15	0.366	0.158	0.365	0.115	0.319
POP2	0.151	0.358	0.25	0.444	0.16	0.367	0.141	0.348
POP3	0.266	0.442	0.1	0.308	0.249	0.433	0.286	0.452
POP4	0.202	0.402	0.3	0.47	0.203	0.403	0.198	0.398
POP5	0.247	0.431	0.2	0.41	0.231	0.422	0.261	0.44
North	0.51	0.5	0.55	0.51	0.525	0.5	0.497	0.5
Center	0.224	0.417	0.2	0.41	0.231	0.422	0.22	0.415
Mezzogiorno	0.265	0.442	0.25	0.444	0.244	0.43	0.282	0.45
Female	0.235	0.424	0.3	0.47	0.219	0.414	0.245	0.431
Ldeaths	5.429	0.46	5.306	0.455	5.458	0.445	5.411	0.471
VarConUnempl	11.398	9.233	11.817	8.818	11.549	9.49	11.267	9.058
# Obs	1025		20		438		567	

**Table 5:** Zero inflation beta regression for subjective probability of losing your job

	(1)	(2)	(3)
	ZOIB	Beta	Zero inflation
	b/se	b/se	b/se
Opinion	0.012* (0.006)	0.011 (0.008)	− 0.021^+^ (0.012)
RedIncome	0.080*** (0.015)	0.058** (0.023)	− 0.168*** (0.034)
Make-ends-meet	0.052** (0.016)	0.063** (0.024)	− 0.071* (0.034)
LHMIncome	− 0.020 (0.015)	− 0.049* (0.024)	− 0.009 (0.029)
WGF19	0.009 (0.024)	0.064^+^ (0.033)	0.068 (0.053)
WGF20	0.003 (0.021)	− 0.044 (0.029)	− 0.074 (0.048)
Support20	0.016 (0.027)	− 0.006 (0.042)	− 0.061 (0.053)
Bonusbb20	0.025 (0.027)	0.002 (0.040)	− 0.075 (0.064)
Remotework	0.020 (0.016)	− 0.023 (0.024)	− 0.097** (0.035)
Ldeaths	0.051* (0.020)	0.064* (0.031)	− 0.069 (0.044)
VarConUnempl	0.001 (0.001)	0.000 (0.002)	− 0.002 (0.002)
Job features	Yes	Yes	Yes
Individual and household features	Yes	Yes	Yes
City size	Yes	Yes	Yes
Macro region	Yes	Yes	Yes
*N*	1005	438	567

The first column shows average marginal effects (AME) for the entire model; the second column shows AME for proportion ranging between zero and one (0,1) estimated through a beta regression; the third column shows AME for the perceived probability of losing the job equal to zero (Logit). In the last column, perceived probability of losing the job has an opposite sign because the logit model takes value 1 as the probability is equal to zero. Robust standard error in parentheses account for the survey weights, p-value: ****p* < *0.001, **p* < *0.01, *p* < *0.5,* ^+^*p* < *0.1*

**Table 6:** Average marginal effects of zero inflation beta regression for subjective probability of losing the job

	(1)	(3)	(4)
	ZOIB	Beta	Zero inflation
	b/se	b/se	b/se
Opinion	0.012* (0.006)	0.011 (0.008)	− 0.021^+^ (0.012)
RedIncome	0.079*** (0.015)	0.056* (0.022)	− 0.168*** (0.034)
Make-ends-meet	0.052** (0.016)	0.063** (0.024)	− 0.071* (0.034)
LHMIncome	− 0.017 (0.015)	− 0.046* (0.023)	− 0.014 (0.029)
WGF19	0.008 (0.024)	0.065* (0.033)	0.070 (0.053)
WGF20	0.003 (0.021)	− 0.046 (0.029)	− 0.079 (0.048)
Support20	0.017 (0.027)	− 0.004 (0.042)	− 0.060 (0.053)
Bonusbb20	0.027 (0.027)	0.003 (0.040)	− 0.080 (0.064)
Remotework	0.008 (0.023)	− 0.054 (0.036)	− 0.106* (0.050)
Ldeaths	0.051* (0.021)	0.061* (0.031)	− 0.070 (0.044)
VarConUnempl	0.001 (0.001)	0.000 (0.002)	− 0.002 (0.002)
Job features	Yes	Yes	Yes
Individual and household features	Yes	Yes	Yes
City size	Yes	Yes	Yes
Macro region	Yes	Yes	Yes
*N*	1005	438	567

The first column shows average marginal effects (AME) for the entire model; the second column shows AME for proportion ranging between zero and one (0,1) estimated through a beta regression; the third column shows AME for the perceived probability of losing the job equal to zero (Logit). In the last column, perceived probability of losing the job has an opposite sign because the logit model takes value 1 as the probability is equal to zero. Robust standard error in parentheses account for the survey weights, p-value: ****p* < *0.001, **p* < *0.01, *p* < *0.5,* ^+^*p* < *0.1*

## Footnotes

1 https://www.bancaditalia.it/statistiche/tematiche/indagini-famiglie-imprese/indag-straord-famiglie-taliane/index.html?com.dotmarketing.htmlpage.language=1.

2 The procedure was implemented using STATA 16 using the zero one inflated beta regression (ZOIB) command available at https://ideas.repec.org/c/boc/bocode/s457156.html.

3 The results are available on request.

4 We are very grateful to the anonymous reviewer who provided us with several useful suggestions*.*
